# A Retrospective Analysis of Lessons Learned and Perspectives on Expansion of Verbal Autopsy Implementation in Zambia, 2023

**DOI:** 10.4269/ajtmh.24-0302

**Published:** 2024-11-12

**Authors:** Stephen Longa Chanda, Mweene Cheelo, Chomba Mwango, Peter Moyo, Kashala Kamalonga, Priscilla Kapombe, Vesper Chisumpa, Emmanuel Tembo, Muzala Kapina, Roma Chilengi

**Affiliations:** ^1^ Surveillance and Disease Intelligence, Zambia National Public Health Institute, Lusaka, Zambia;; ^2^ Department of Public Health, Ministry of Health, Lusaka, Zambia;; ^3^ Bloomberg Data for Health Initiative, Lusaka, Zambia;; ^4^ Department of National Registration Passports and Citizenship, Ministry of Home Affairs and Internal Security, Lusaka, Zambia;; ^5^ U.S. Centers for Disease Control and Prevention, Lusaka, Zambia;; ^6^ School of Humanities and Social Science, Department of Population Studies, University of Zambia, Lusaka, Zambia;; ^7^ Director General, Zambia National Public Health Institute, Lusaka, Zambia

## Abstract

Accurate cause-of-death statistics are vital for public health policy, but less than one-third of deaths globally are assigned a cause. Verbal autopsy (VA) methods are crucial in low- and middle-income countries lacking complete civil registration and vital statistics (CRVS) systems. We explored VA implementation in Zambia by using a previously developed framework. The National Mortality Surveillance Subcommittee under the Monitoring and Evaluation Technical Working Group within the Ministry of Health coordinates mortality surveillance activities in Zambia. To date, passive, non-population-representative VA data collection mechanisms have been used, leading to underrepresentation of some communities. In spite of the use of electronic data collection tools, VA systems have not been electronically linked to public health surveillance or CRVS systems. Funding for VA has largely been donor driven. Increasing government funding may ensure sustainability, while the adoption of sample-based platforms while linking VA information technology systems may make VA data more useful, timely, and accessible.

## INTRODUCTION

Accurate and population-representative cause of death (COD) statistics are vital for informing public health policy, addressing emerging health needs, tracking progress towards Sustainable Development Goals, and achieving national developmental milestones.^1^^–^^3^ However, globally, less than one-third of deaths are assigned a cause, with even fewer in Africa.^3^ The ideal source of population-representative COD statistics for mortality surveillance is a complete, universal, and continuous civil registration and vital statistics (CRVS) system.^4^^,^^5^

However, in many low- and middle-income countries (LMICs), CRVS systems struggle to provide timely, complete, and accurate vital statistics, with less than 20% of estimated deaths registered annually in Zambia.^6^^,^^7^

The gold standard procedure for determining COD is a complete diagnostic autopsy (CDA).^8^

For the subset of deaths that occur in health facilities where a CDA is neither available nor affordable, medical certification of COD (MCCOD) is conducted using all patient records and other medically relevant information about the terminal illness.^8^ However, in settings where CDAs or MCCODs are rare and cultural or infrastructural constraints exist, the WHO recommends the use of verbal autopsy (VA) methods.^9^ Verbal autopsy involves structured interviews with family members or caregivers of the deceased to ascertain probable causes of death using either physicians or computer algorithms.^9^ Despite limitations at the individual level, VA remains a crucial method for assigning COD among community deaths in LMICs lacking fully developed CRVS systems.^7^^,^^10^^–^^12^

**Figure 1.**
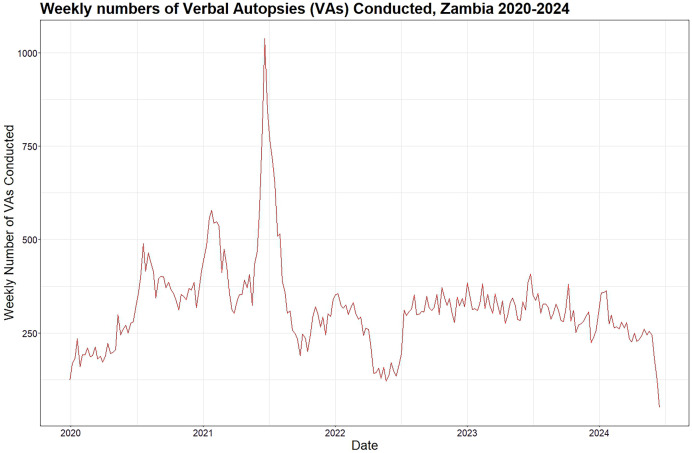
Trendline of the weekly number of verbal autopsies conducted in Zambia, January 2020 to June 2024.

**Figure 2.**
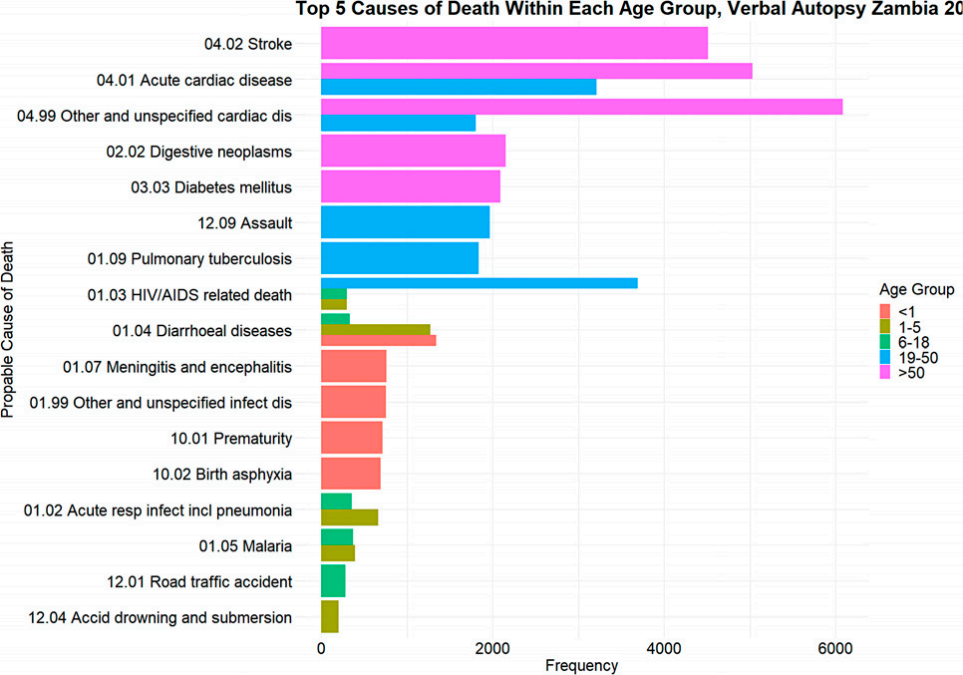
Frequency of the top five probable causes of death within each age group, Zambia verbal autopsy, 2020–2024.

In Zambia, VA has been successfully implemented in research settings, notably in the Sample Vital Registration with Verbal Autopsy (SAVVY) project, demonstrating the feasibility of collecting vital statistics using standardized methods. This was conducted as a pilot study in four provinces of Zambia from 2010 to 2011 and established that collecting vital statistics using standardized SAVVY methods was feasible in Zambia.^13^ Additionally, the Ministry of Home Affairs and Internal Security Department of National Registration Passports and Citizenry (DNRPC) and the Ministry of Health (MOH) have conducted VAs as part of routine death registration processes. Between January 2017 and September 2020, DNRPC’s implementation aimed to complement civil registration.^11^^,^^13^^–^^15^ This was implemented across eight sites in six districts of Zambia. This implementation was coordinated by DNRPC with technical assistance from the Bloomberg Data for Health Initiative (D4H) and the CDC Foundation. From 2019 to date, MOH’s expanded VA initiative across 30 sites in 23 districts supported by the President’s Emergency Plan for AIDS Relief through the United States Centre for Disease Control and Prevention-Zambia (U.S. CDC-Zambia) targeted the monitoring of HIV-associated mortality and improving death registration (Figures 1 and 2; Table 1).

**Table 1:** Demographic characteristics of verbal autopsies done in Zambia, January 2020–June 2024

Characteristics	*N* = 74,454	
	*n*	%
Sex		
Female	30,806	41.0
Male	43,640	59.0
Unknown	8	<0.1
Age, years, median (IQR)	46	(26.0–70.0)
Unknown	25	–
Age group		
<1	6,462	8.7
1–5	4,080	5.5
6–18	3,821	5.1
19–50	26,951	36.0
>51	33,115	44.0
Unknown	25	–
Cause of death*		
Acute cardiac disease	8,419	11.0
Other and unspecified cardiac disease	8,174	11.0
HIV/AIDS-related death	6,339	8.5
Stroke	5,313	7.1
Acute respiratory infection, including pneumonia	4,721	6.3
Diarrheal diseases	4,615	6.2
Digestive neoplasms	3,675	4.9
Pulmonary tuberculosis	3,216	4.3
Indeterminate	2,967	4.0
Diabetes mellitus	2,718	3.7
Other^†^	24,297	33.0

IQR = interquartile range.

* Top 10 causes of death as ascribed by the InterVA-5.

^†^ All other probable causes of death assigned by the algorithm.

In 2022, the Zambia National Public Health Institute (ZNPHI) launched the country’s routine mortality surveillance program through the establishment of a mortality surveillance unit at ZNPHI. This program is designed to enhance mortality surveillance in Zambia by bringing together various stakeholders and scaling up the existing sentinel VA to a broader, population-level application. We apply the framework developed by de Savigny et al.^3^ to describe current VA implementation in Zambia and to develop recommendations for its improvement. This framework outlines the necessary system-level components for effective VA implementation, which include governance, design, operations, human resources, financing, infrastructure, logistics, information technologies, and data quality assurance (DQA).^3^

### Governance.

The implementation of the VA in Zambia has, to date, always been research protocol based,^11^^,^^13^^–^^15^ and as such, there has been no national interagency oversight mechanism. In 2022, Zambia established a National Mortality Surveillance Subcommittee under the Monitoring and Evaluation Technical Working Group within the MOH. The subcommittee was created to address gaps in the coordination of mortality surveillance activities in Zambia, to support the routine use of mortality data for public health decision-making, and to bolster ongoing efforts to improve death registration within the Zambian CRVS. This subcommittee comprises members from key government ministries, departments, and institutions, nongovernmental organizations, and academia (Table 2).

**Table 2:** Members of the Zambia Mortality Surveillance Subcommittee and their respective roles

Stakeholder	Roles and Responsibilities
MOH	MOH works towards electronic notification of deaths and facilitates paper-based notification of deaths during the verbal autopsy.
DNRPC	DNRPC provides technical support to staff conducting death notification and verbal autopsy and provides/collects death notification forms. DNRPC working with the Ministry of Local Government and Rural Development and the MOH ensures registration of all deaths, including those that take place in rural areas and away from burial sites under local council jurisdiction.
Ministry of Local Government and Rural Development	Ensures the coordination of death registration and notifications from Local Councils and from the traditional leadership through the House of Chiefs
ZAMSTATS	Is responsible for the analysis and dissemination of information generated from mortality surveillance at the national and subnational levels
ZNPHI	Collaborates with DNRPC and ZAMSTATS to analyze and map mortality trends and patterns and shares this information with the other members. Any shifts from normal/expected trends are investigated and reported on by ZNPHI. ZNPHI additionally coordinates all the other stakeholders.
The University of Zambia	Leads in ensuring that information generated from the mortality surveillance program is used for research purposes. The university will ensure that results stemming from research are available and can be used for informed policy-making decisions.
The World Health Organization County office	Provides technical support
U.S. CDC-Zambia	Provides technical and financial support
Bloomberg Data for Health Initiative	Provides technical and financial support

DNRPC = Department of National Registration Passports and Citizenry; MOH = Ministry of Health; ZAMSTATS = Zambia Statistics Agency; ZNPHI = Zambia National Public Health Institute.

### Design, operations, information technology (IT), infrastructure, logistics, and human resources.

Purposive sampling was used to select districts for inclusion in both systems. In the DNRPC system, sites were chosen based on districts exhibiting high death registration completeness and well established death notification systems. In the MOH system, district selection was limited to provinces where the U.S. CDC-Zambia was currently implementing activities. Within these provinces, high HIV morbidity and mortality served as key inclusion criteria for selecting districts for VA implementation. Both systems used passive data collection methods, relying on the decedent’s next of kin to report the death while accessing burial services. Potential interviewees were identified among mourners as they brought the deceased to the health facility mortuary. At most sites, VA interviewers have dedicated office space but often share it with mortuary attendants or DNRPC officers stationed at health facilities. Verbal autopsy was promptly conducted after the appropriate respondent was identified at the health facility mortuary as the family accessed burial services. The MOH has been implementing tele-VAs to a limited extent, using phone calls to conduct VA interviews remotely.

During data collection, DNRPC initially piloted the electronic SmartVA Questionnaire system,^16^ paper based, and then later transitioned to the WHO 2016 Questionnaire^17^ on the Open Data Kit (ODK)^18^ platform. The D4H provided technical support in system design and questionnaire translation, whereas DNRPC provided IT support staff and systems for transmission and storage through the government-wide area network. Conversely, the MOH VA system began with ODK and is currently transitioning to a KOBO-based data collection platform.^19^ Both electronic systems transmitted completed VA forms to an online server, which applied an algorithm to assign a probable cause of death (SmartVA Analyze^20^ and Inter-VA-4^21^ [DNRPC] and Inter-VA-5^22^ [MOH]). These different algorithms are available on the openVA platform, the first platform to offer a standardized framework for analyzing VA data, ensuring compatibility with all publicly available methods and data structures.^23^ To a limited extent, DNRPC used physician-coded VAs to assign a cause of death. The VA questionnaire was locally adapted and translated into the three most widely spoken local languages (Bemba, Tonga, and Nyanja) in the sentinel sites.

Regarding human resources, full-time project staff with a minimum qualification of a grade 12 General Certificate of Education were hired to serve as VA interviewers by both the MOH and DNRPC. The DNRPC recruited and trained 25 interviewers and a data manager. Meanwhile, the MOH established several full-time project positions for VA implementation: HIV-associated mortality surveillance advisor (1), mortality surveillance epidemiologist (1), mortality surveillance data manager (1), field mortality surveillance officers (55 VA interviewers), and a driver (1). After recruitment, VA interviewers underwent training before field deployment, with subsequent refresher training as needed. The training curriculum for VA interviewers in both the DNRPC and the MOH included the following topics: an overview of VA, the roles of VA interviewers, the WHO 2016 VA questionnaire (covering neonates, children, and adults), interview techniques, ethics, and VA administration using tablets.

Despite the use of electronic data platforms for collecting, transmitting, and analyzing VA data, there were no connections between these IT systems and other electronic health/civil registration platforms. This lack of linkage has hindered the timely release of VA data for public health surveillance and civil registration purposes. As both systems relied on passive data collection from large referral health facilities, deaths occurring within rural communities were underrepresented in both systems. Furthermore, because study sites were nonrandomly selected, neither system generated population-representative VA data. These findings underscore the necessity for study designs that encompass national as well as subnational representativeness and highlight the importance of community-based surveillance platforms capable of capturing all deaths, regardless of location.^24^^–^^26^

### Financing.

The primary source of funding for the implementation of VA in Zambia has been donor support, supplemented by funding from the Government of the Republic of Zambia (GRZ). In the DNRPC implementation, donor support came from D4H, the U.S. CDC-Zambia/CDC Foundation, and the Global Fund, providing financial and technical assistance. In the MOH implementation, U.S. CDC-Zambia provided financial and technical aid through the U.S. President’s Emergency Plan for AIDS Relief, whereas the CDC Foundation, through D4H, offered technical assistance. The GRZ, through the MOH and DNRPC, has supported VA implementation through infrastructure and human resources. We could not calculate the average cost per VA in both systems due to a lack of information. However, the main cost drivers in both systems were training and retraining project staff, procuring IT infrastructure, and remunerating VA interviewers and their supervisors.

### Data quality assurance.

During the DNRPC implementation, there was no routine DQA. However, in the MOH system, DQA occurs in two phases, during data collection and through on-site data audits. During data collection, the ODK/KOBO platform incorporates internal checks to ensure the consistency of the entered information. Once collected, these data are transmitted directly to the national server, where both automated and manual data checks are conducted. The on-site data audits are conducted using a structured tool. This tool compares the number of VAs in the Verbal Autopsy Explorer,^27^ an online dashboard tracking conducted VAs, with the number of community deaths recorded in mortuary registers and the District Health Information System-2. There is currently no monitoring and evaluation framework in place to oversee this implementation.

### Summary and recommendations for population-representative VA in Zambia.

The implementation of VA in Zambia has improved mortality surveillance by providing timely information on causes of death among community deaths using electronic data capture and cause-of-death determination tools. Achievements include successful pilot projects such as the SAVVY, expanded VA initiatives across multiple districts (DNRPC and MOH implementation), and substantial technical and financial support from donors and the government. Better coordination of mortality surveillance strengthening activities has been achieved through the National Mortality Surveillance Subcommittee. However, limitations include the underrepresentation of rural areas, lack of electronic integration with CRVS and public health systems, non-population-representative data due to purposive sampling, and a heavy reliance on donor funding.

To better coordinate mortality surveillance activities in Zambia, we propose establishing a dedicated interministerial Mortality Surveillance Coordinating Committee. This committee would oversee the expansion of VA initiatives and the establishment of vital connections with the CRVS with technical support from ZNPHI’s mortality surveillance unit. Given the historical reliance on donor support for funding VA initiatives in Zambia, it is imperative to explore avenues for gradually increasing funding through government mechanisms. Sustainable financing strategies will be essential for maintaining the long-term viability of VA programs and ensuring their continued impact on public health.

To address the need for population-representative VA data, we suggest adopting sample-based systems for VA data collection. This approach will help ensure a more comprehensive understanding of mortality patterns across different regions of Zambia. To facilitate the timely collection and utilization of VA data, we recommend integrating electronic data collection and analysis platforms with other electronic public health response and CRVS systems. Further customization of the VA questionnaires into more local languages is required to promote inclusivity and adapt to the diverse linguistic landscape of Zambia (Table 3).

**Table 3:** Comparison of various elements of the verbal autopsy systems implemented by the Ministry of Home Affairs and Internal Security and the Ministry of Health, Zambia 2016–2023

Area	MOHAIS	MOH	Recommendation for Future State
Governance and finance	Project based (D4H, U.S. CDC-Zambia/CDC Foundation, Global Fund)	Project based (PEPFAR, U.S. CDC-Zambia, CDC Foundation through the D4H)	Use the project base to advocate for greater government funding
Design	Non-population-representative sample; purposive sampling in 7 sites	Non-population-representative sample; purposive sample selected for high HIV burden districts; 30 sites	A nationally and subnationally representative population-based sample
Operations	Passive system	Passive system	Active and passive system
Human resources	Project staff	Project staff	Project staff and Government of the Republic of Zambia employees across key ministries involved in mortality surveillance
IT and data quality assurance mechanisms	Electronic data management system	Electronic data management systems	Electronic data management systems and system integration between data producers and data users

D4H = Bloomberg Data for Health Initiative; IT = information technology; MOH = Ministry of Health; MOHAIS = Ministry of Home Affairs and Internal Security; PEPFAR = President’s Emergency Plan for AIDS Relief; U.S. CDC-Zambia = United States Centre for Disease Control and Prevention-Zambia.
